# A bioinformatics expert system linking functional data to anatomical outcomes in limb regeneration

**DOI:** 10.1002/reg2.13

**Published:** 2014-07-10

**Authors:** Daniel Lobo, Erica B. Feldman, Michelle Shah, Taylor J. Malone, Michael Levin

**Affiliations:** ^1^ Center for Regenerative and Developmental Biology and Department of BiologyTufts University200 Boston Avenue, Suite 4600MedfordMA02155U.S.A.

**Keywords:** Limb regeneration, ontology, bioinformatics, database, developmental biology, expert system, arthropod, amphibian, crustacean

## Abstract

Amphibians and molting arthropods have the remarkable capacity to regenerate amputated limbs, as described by an extensive literature of experimental cuts, amputations, grafts, and molecular techniques. Despite a rich history of experimental effort, no comprehensive mechanistic model exists that can account for the pattern regulation observed in these experiments. While bioinformatics algorithms have revolutionized the study of signaling pathways, no such tools have heretofore been available to assist scientists in formulating testable models of large‐scale morphogenesis that match published data in the limb regeneration field. Major barriers to preventing an algorithmic approach are the lack of formal descriptions for experimental regenerative information and a repository to centralize storage and mining of functional data on limb regeneration. Establishing a new bioinformatics of shape would significantly accelerate the discovery of key insights into the mechanisms that implement complex regeneration. Here, we describe a novel mathematical ontology for limb regeneration to unambiguously encode phenotype, manipulation, and experiment data. Based on this formalism, we present the first centralized formal database of published limb regeneration experiments together with a user‐friendly expert system tool to facilitate its access and mining. These resources are freely available for the community and will assist both human biologists and artificial intelligence systems to discover testable, mechanistic models of limb regeneration.

## Introduction

The regeneration of amputated limbs is one of the most striking examples of the restoration of a functional and complex structure (Birnbaum and Alvarado 2008). It is still largely unknown why some animals can regenerate large sections and complete appendages while others have a very limited regenerative capacity (Brockes and Kumar 2008). Organisms such as salamanders, frog tadpoles, and molting arthropods can regenerate complete limbs after amputation at any level (Maruzzo et al. 2005; Stocum and Cameron 2011; King et al. 2012). Understanding the mechanisms that permit these animals to restore lost limbs will pave the way for applications in the context of regenerative medicine to address human injury (Whited and Tabin 2009; Levin 2011). To this end, the field of regenerative biology has produced an extremely rich dataset of experiments based on classical surgical methods together with new molecular techniques and genetic tools. These various perturbations of the regenerative process result in a vast variety of morphological outcomes. Salamander limbs having multiple hands (Tank 1981), crab chelipeds composed of multiple claws (Nakatani et al. 1998), and cockroach limbs with intercalary multiple regenerates (French 1976a) are typical experimental results found in the literature.

Despite this large and ever‐growing dataset of regenerative limb experiments, there is no comprehensive mechanistic model that can explain how the correct (or incorrect) limb patterning occurs (Nacu and Tanaka 2011); the signaling mechanisms that regulate the process in which an amputated limb regenerates exactly the missing parts are unknown (Bryant et al. 2002). The latest advances in molecular pathways are identifying genes and molecules necessary in the regenerative process: molecular interference with certain elements causes a malfunction in the regenerative mechanism (Stocum and Cameron 2011). However, this is far from a comprehension of how regeneration is accomplished. Indeed, we need to identify the *sufficient* mechanisms that allow the repairing and regrowth of a missing limb. This profound disconnect between high‐resolution data at the molecular level and large‐scale anatomical outcomes is holding back the development of biomedical applications to control and translate the outstanding regenerative capacity that these organisms possess. Progress on the human biomedicine of limb injury requires that we formulate algorithmic models that unambiguously show how overall limb shape results from cell behaviors, because these will most clearly indicate where perturbations can be applied to trigger specific patterning events.

One of the main reasons for the lack of comprehensive models of limb regeneration is precisely the huge amount of raw functional and molecular resources found in the literature. Although great efforts in sequencing genetic information have produced important centralized transcriptomic, genomic, and epigenetic datasets on limb regeneration (Habermann et al. 2004; Putta et al. 2004; Durica et al. 2006; Guerrero et al. 2006), the field currently lacks any tools for mining the enormous published dataset on the most important aspect of regeneration: the endpoint – repair of large‐scale anatomical shape. The absence of formal analytical tools for functional data causes the paradox that, the larger the dataset becomes, the less we understand the studied system (Lazebnik 2002). Indeed, we have surpassed the threshold where a single person can keep track of all the information distributed in all the papers in the field; students entering this fascinating field are frustrated that no single repository exists which can be queried to determine what experiments have been done and what the outcomes of a specific perturbation were. Moreover, the data are now so rich that it is all but impossible for human experts to propose mechanistic models whose predictions match even a basic subset of the known results. Lastly, the emergent systems‐level pattern formation arising from cells following simple rules is often highly nonlinear and complex. The need to store and mine results of the regeneration literature, as well as the need to simulate models to determine their patterning properties, both converge on an unavoidable fact: the necessity of extending bioinformatics tools beyond molecules to morphology, so that computational tools can help human scientists discover models whose behavior fits the known facts of regeneration (Lobo et al. 2012). Here we take the first essential step of this fundamental roadmap and produce a database that will be used by computational tools and human scientists to check their models’ predictions against the known results of morphological experiments. Each hard‐won model formulated by investigation of gene pathways is valuable to the extent that it predicts, explains, and helps control patterning processes. Our system connects the products of molecular and cell biology research with the known patterning properties of regeneration, enabling a new generation of tools for testing biochemical and genetic models against the existing published data.

A method to unambiguously encode biological shape is an essential part in a formalization of regenerative science. Natural language is imprecise and ambiguous (King et al. 2011), and a formal descriptive method is necessary to achieve semantic clarity. Ontologies define formal rules and logical connections to structure the knowledge of a particular domain (Bard 2003). They have extensively been applied to formalize scientific data in several biological fields, especially genetics (Soldatova and King 2005, 2006; Whetzel et al. 2006; Visser et al. 2011). Ontologies have also been applied to formalize phenotype data (Robinson and Mundlos 2010; Schindelman et al. 2011) and have been used in structured databases linking genetic and phenotypic data (Groth et al. 2007; Eppig et al. 2012; Yook et al. 2012). However, these ontologies describe phenotypes with predefined attributes, such as “enlarged heart” or “pericardial edema” (Smith and Eppig 2009), or associate predefined entities (“eye”, “tail”) with predefined qualities (“small”, “round”) (Beck et al. 2009; Washington et al. 2009; Mungall et al. 2010). Although attribute‐based ontologies are a great improvement over natural language descriptions, they are not adequate for the formalization of regenerative appendages. A regenerated limb consists of one of many possible topological and geometrical relationships between the segments that compose the morphology. For example, grafting experiments can result in limb morphologies with two or three hands, each of them with a variable number of digits, that may be located in the wrist or ectopically in the elbow. At the same time, highly detailed morphometric data can often obscure the important aspects of a morphological outcome among rich quantitative measurements of irrelevant or highly variable properties of individual animals.

Previously, we designed and successfully applied a new kind of mathematical ontology for the formalization of planarian regenerative experiments (Lobo et al. 2013a,b). This ontology overcomes the challenge of representing regenerative phenotypes by using mathematical graphs. Mathematical graphs have been extensively used for modeling and analyzing pattern representations (Conte et al. 2004) and biological phenomena (Mason and Verwoerd 2007) including morphological data (Nagl 1979; Doi 1984; Lobo and Vico 2010; Bard 2011; Lobo et al. 2011). In contrast to attribute ontologies, a mathematical graph can represent the topological relations between parts, as well as their geometrical and spatial relations (Pach 1999). In addition, a mathematical formalization of regenerative phenotypic data can be extended to formalize manipulation data. Cuts, amputations, and grafts and their combinations are difficult to express in natural language. In contrast, specific mathematical formalisms can be used to precisely and unambiguously define the complex manipulations typically used in regenerative experiments (Lobo et al. 2013b). This new kind of ontology based on mathematical graphs is an ideal approach for the formalization of limb phenotypes and manipulations.

Here, we report a novel ontology system for the field of limb regeneration. We designed an intermediate, symbolic representation based on graphs that highlights the key features of anatomy and facilitates comparison between experiments. Our optimal level of abstraction allowed us to include several very diverse model species in this analysis − a must if deep, general principles of shape regulation are ever to be discovered. Based on this novel ontology, we created an expert system database and query tool for this rich field. This system is the first, essential step in the effort of enabling powerful artificial intelligence tools to mine functional data and derive testable models explaining them.

## Results

The knowledge base for the expert system on limb regeneration must contain all of the experimental perturbations performed in the past experiments, with their corresponding patterning outcomes. The first step for any knowledge base approach (for human or computerized analysis) is to define a formalism in which to describe the data. In order to fill a database with the published results in this field we defined formal descriptors of limb morphologies and of experimental actions.

### Formalism for limb morphologies

In order to unambiguously characterize the morphology of limbs of regenerative organisms, we propose a mathematical formalism based on labeled graphs. A graph is a formal representation of a set of interconnected objects, represented with vertices and edges (links between vertices). The proposed formalization follows the natural division of a limb morphology into segments: each limb segment is represented by a graph edge, and edges are interconnected by joints represented as graph vertices. In this way, edges and vertices represent the segments and their joints in a limb morphology. The geometric characteristics of the limb morphology are stored as labels in the vertices and edges. The formalism is powerful enough to be applied to different types of limbs from a wide set of species, which we have grouped into different categories. Figure 1 shows a wild‐type morphology of a representative organism for each category currently present in the database along with its formalization represented as a schematic diagram and a cartoon. These categories currently include salamanders, frogs, crustaceans, insects, and arachnids (Fig. 1A−E, respectively).

**Figure 1:**
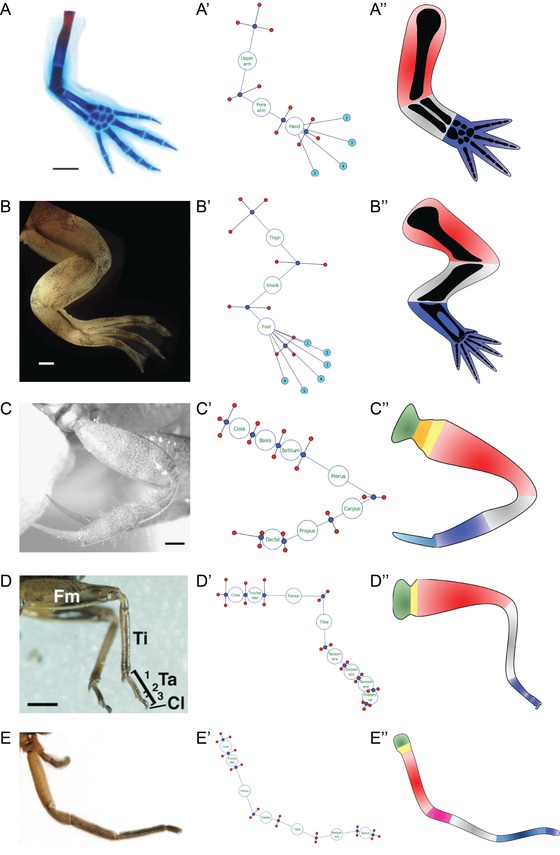
Wild‐type limb morphologies from different regenerative organisms and their representation with the proposed formalism. (A)−(E) Wild‐type morphology of a representative organism of each category currently present in the database, including salamander (A, axolotl forelimb), frog (B, *Xenopus* hindlimb), crustacean (C, fiddler crab limb), insect (D, cricket nymph limb), and arachnid (E, huntsman spider limb). (A′)−(E′) Graph representation of the formalization of the wild‐type morphologies in A−E, respectively. The formalization represents limb morphologies by dividing them into segments (labeled edges) and joints between segments (blue dots). Parameters store the basic shape (red dots) and the location, length, and type of digits (cyan dots). (A′′)−(E′′) Cartoon diagrams automatically generated from the formalized data represented in A′−E′, respectively. The overall shape representation and color‐coded segments show a clear and unambiguous depiction of the encoded limb. Scale bars 1 mm. (A)−(E) are adapted from Whited et al. (2013), Yokoyama et al. (2011), Stueckle et al. (2008), Nakamura et al. (2008b), and Agnarsson and Rayor (2013), respectively.

Graph edges (blue lines with a center label, Fig. 1A′−E′) represent limb segments, and they are labeled with the type of segment that they represent. Each category contains a set of limb segments characteristic of that group of organisms. For example, the salamander and frog forelimbs include upper arm, fore arm, and hand segments (Fig. 1A′), whereas their hindlimbs include thigh, shank, and foot segments (Fig. 1B′). In contrast, crustaceans have a larger set of limb segments, including coxa, basis, ischium, merus, carpus, propus, and dactyl (Fig. 1C′). In addition to the segment type, a graph edge is labeled according to its geometrical parameters (distance and angle between its vertices), its side (left or right), and whether it is reversed (a 180° rotation around the proximal−distal axis) or inverted (swapping the natural proximal and distal segment ends). Segment nerves can be defined in the formalism by a sequence of consecutive locations outlining the path of the nerve. Finally, salamander and frog limb categories can include for each segment the number of bones and digits that are present. Digits are specified according to their position with respect to the segment they are attached to and the number of bones, including metacarpal or metatarsal bones and phalanges (cyan dots, Fig. 1A′, B′).

Graph vertices represent joints between segments (blue dots, Fig. 1A′−E′), and can be connected to one, two, or more edges. The length of a segment is defined as the distance between its two joints and is stored as an edge label, while segment shapes are abstracted as a list of numerical parameters and stored as a vertex label. The shape parameters represent the distance between the center of the joint and the limb morphological border in a specific direction (red dots, Fig. 1A′−E′). Joints have a parameter per each bisector of every two consecutive segments, except joints connected to a single segment, which have three parameters corresponding to the fixed angles of +90°, +180°, and +270° with respect to the direction of the segment. In this way, the size and shape of a segment are defined by a combination of the segment length parameter and its joints’ shape parameters. Therefore, the formalism can encode limbs with similar shape but different size (same joint shape parameters but different segment length parameters) and limbs with similar size but different shape (same segment length parameters but different joint shape parameters).

The formalism can encode any limb morphological configuration. Any segment topology can be represented, since a graph can decompose the plane (or a volume) in any arbitrary configuration. This property makes the formalism complete (universal): all possible wild‐type and experimental morphologies are representable. A limb morphology graph is always connected (a path always exists between any two segments). Wild‐type morphologies can always be represented by linear graphs (Fig. 1), where the most proximal and distal vertices connect to one segment and the in‐between vertices connect two edge segments. In contrast, grafting experiments can result in limbs with three or more segments attached to the same vertex, or a segment attaching directly to another segment. Figure 2 shows a selection of encoded limb morphologies that result from experiments found in the scientific literature, including limbs with arbitrary ectopic structures, such as digits, hands, or full limbs. In addition to the morphological configuration, the formalism can accommodate the shapes of the segments of a multitude of structures, such as crustacean chelipeds (as a combination of propus, fixed finger, and dactyl segments) or insect claws (pretarsus segments).

**Figure 2:**
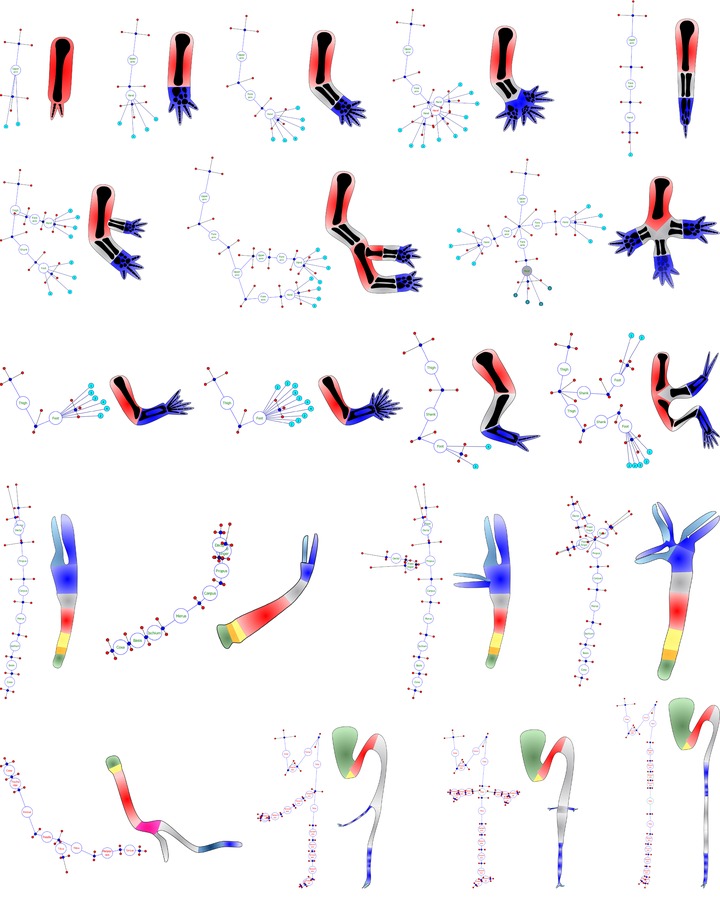
Selection of formalized limb morphologies included in the database and represented by graph and cartoon diagrams. The different types of limb segments are easily differentiated with color‐coded areas and bone configurations in the case of salamanders and frogs. Morphologies from different categories have a different set of limb segments characteristic of such group. The formalism can also encode inverted and reversed segments.

In addition to the graph diagram to visualize formalized morphologies, we implemented a simple algorithm that automatically draws a cartoon representation given an encoded morphology. Figures 1A′′−E′′ and 2 show automatically generated cartoon illustrations of the corresponding morphologies. Segments are color‐encoded according to their type, and the limb shape and topological configuration are derived from the connectivity and parameters stored in the formal encoding. When appropriate, bone silhouettes are included to aid the identification of different segments. In this way, the most important characteristics of a limb morphology, such as segment configuration and overall shape, are clearly and unambiguously specified and illustrated with the presented formalism.

### Formalism for limb manipulations

Alongside the limb morphology descriptors, we propose a formalism to precisely and unambiguously encode the manipulations performed in a limb regeneration experiment. The techniques most used in this field include surgical, genetic, pharmacological, and irradiation interventions. We have formalized these interventions as the following possible basic actions:

- remove: a section of the limb is cut and discarded, keeping the rest of the limb
- crop: a section of the limb is cut and kept, discarding the rest of the limb
- join: two sections from two different limbs are grafted together
- relocate: a section from a limb is cut and grafted in a different part of the same limb
- reverse: a limb is reversed before being grafted
- irradiate: a section of the limb is irradiated.

When entering an experiment into the database, these basic actions are defined with specific parameters, such as a polygon to define the borders in the case of a “remove” action or the grafting position and angle of rotation in the case of a “join” action. Simple incisions can also be encoded as a “remove” action with two points as parameters, unambiguously defining the location and length of the surgical cut.

Basic actions are usually combined together during the procedure of a typical experiment. The formalism encodes the order of the individual actions in a manipulation using a mathematical tree. A mathematical tree represents a hierarchical graph structure, where the vertices represent a basic manipulation action or the initial limb morphology from which to start the experiment and the edges specify the order of the application of the manipulations. All basic manipulation actions receive an input (a limb to manipulate) and produce an output (a manipulated limb or amputated limb part), except the join action, which receives two inputs (a host limb and a piece to graft to) and produces an output (the limb with the grafted piece). Remove, crop, relocate, and irradiate actions are labeled with a list of spatial points defining a polygon that defines the area of the limb affected. A relocate action, in addition to the cut polygon, is labeled with a displacement vector and rotation angle of the graft coming from the same limb. Similarly, a join action is labeled with a displacement vector and rotation angle of the graft, but, in this case, the graft is obtained from another limb. A mathematical tree can then interconnect these basic actions and specific morphologies (encoded according to the formalism for morphologies presented above) to form any possible complex manipulation for an experiment.

Figure 3 illustrates the formalization of a typical manipulation from a salamander regeneration experiment where an amputated hand is grafted into a cut upper arm. The top two vertices in the figure (the leaves of the tree) specify the starting limbs for the experiment. In this example, the two wild‐type forelimbs of the salamander are used: left (left‐top vertex) and right (right‐top vertex). The manipulation then proceeds by amputating the left limb at the middle level of the upper arm. This basic action is encoded with a remove action (left vertex) that receives the wild‐type left limb as input and defines a polygon (yellow dots) selecting the section to amputate and discard. In addition, the manipulation amputates the hand of the right limb. This is encoded with a crop action (right vertex) that receives the wild‐type right limb as input and specifies with a polygon (yellow dots) the limb section (the hand in this case) to amputate and preserve for the next step. Finally, the manipulation takes the amputated hand from the right limb and grafts it into the amputated upper arm from the left limb. This final action is specified in the formalism with a join action that receives the outputs of the previous remove and crop actions. The join action is labeled with the proper position vector and rotation angle of the graft with respect to the host. The result of the join action (the root of the tree) represents the final configuration of the manipulation, which regeneration outcome will be scored in the corresponding experiment.

**Figure 3:**
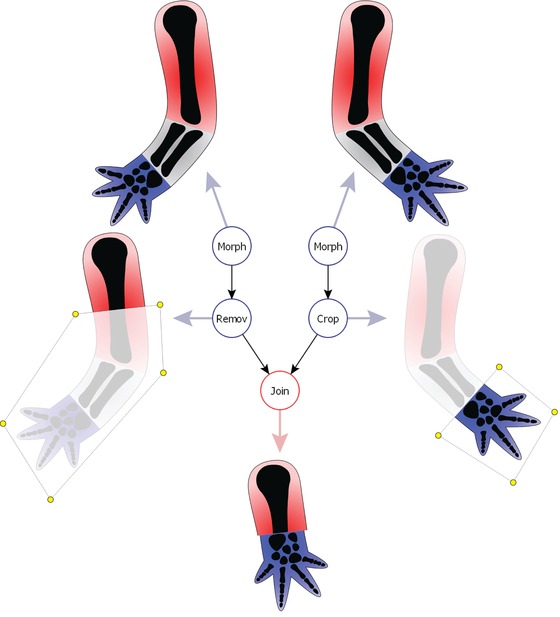
Example of a formalized limb manipulation. A mathematical tree formalizes the steps (vertices of the tree) required to perform a manipulation and their order of execution (edges of the tree). The diagram represents such a formalization, where a hand is amputated (right vertex) from a wild‐type right limb (top‐right vertex) and grafted (bottom vertex) into a wild‐type left limb (top‐left vertex) amputated at the middle level of the upper arm (left vertex).

Figure 4 shows a selection of formalized manipulations curated from the scientific literature. Amputations can be performed to large areas, such as the whole hand, or smaller sections, such as a part of a bone or a single digit. Similarly, grafts transplanted to a limb can comprise several limb segments or just the distal cap of the limb. Irradiation exposures are usually applied to limb parts that are amputated and grafted into a non‐irradiated limb. Nerve manipulations are frequent in amphibian limb experiments, including complete denervation of a major nerve (formalized with remove actions) and major nerve deviations (formalized with relocate actions). In summary, all types of amputations, grafts, and subtle manipulations, including nerve deviations, partial irradiations, and incisions, can be unambiguously encoded with the presented formalism.

**Figure 4:**
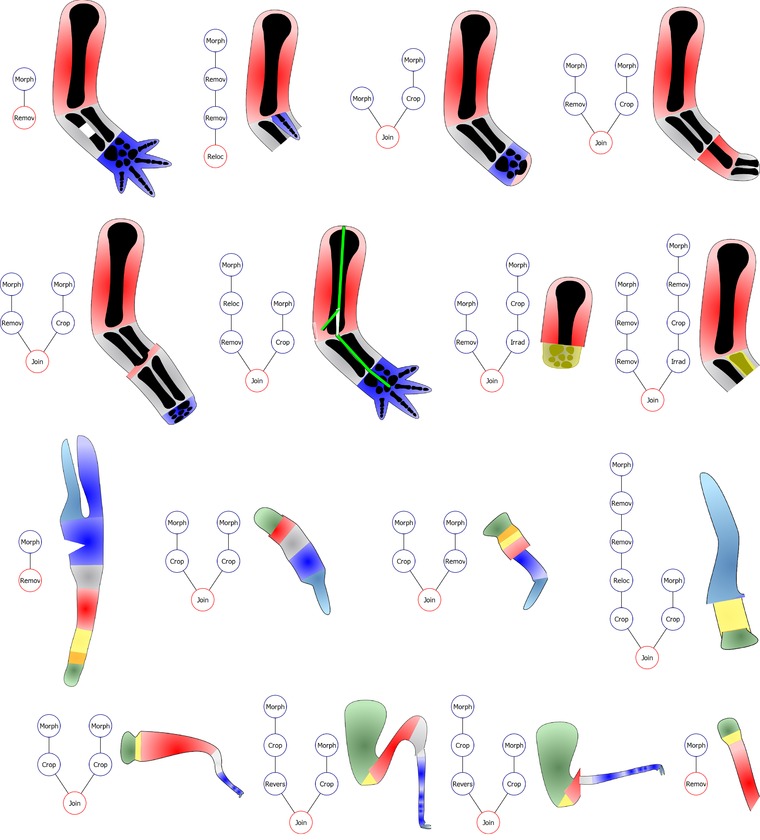
Selection of formalized limb manipulations included in the database. Each manipulation is represented by a mathematical tree diagram and an automatically generated cartoon of the final configuration. The formalism is able to encode practically any type of cut, amputation, grafting, irradiation (yellow areas), and nerve deviation and their combinations.

### Formalism for limb regeneration experiments

The formalization of experimental manipulations and their resulting morphologies are combined together to create a complete formalism to describe any regenerative experiment. A specific experiment from a published study is then described with its relevant details, including a manipulation and a set of morphological outcomes. In the database, an experiment is encoded with the presented formalism with the following information: a descriptive unique name, the publication describing the results, the organism species, any treatments used (such as pharmacological compounds in the medium or injected), the formalized manipulation, and the formalized resultant morphologies.

A manipulated limb can present different phenotypes at different regeneration times. In addition, different animals can regenerate different phenotypes with the same treatment, resulting in different phenotypes at the same regeneration time (incomplete penetrance). Accordingly, the formalism can capture multiple resultant phenotypes for a single experiment, due to different regeneration times or treatments with incomplete penetrance. In particular, an experiment in the formalism includes a set of morphologies grouped by the time period at which they appear since the manipulation time‐point. For each recorded time period, the formalized experiment stores the total number of animals used (*N*) and the frequency distribution of each resultant morphology. In this way, formalized experiments can encode any combination of phenotypes at multiple regeneration times. Published experiments reporting only aggregated data (e.g., the total number of segments regenerated in a set of individuals) instead of the frequency of the resultant phenotypes are listed in the database with a label called “Aggregated data” to indicate that the frequencies are not exact but estimated.

### Database of limb regeneration experiments

We modeled and implemented the presented formalism for limb experiments in a relational database. Figure 5 shows a diagram of the database schema, including the tables and their logical relations as defined in the database. The database is organized around the organism categories (center), which currently contains entries for salamander, frog, crustacean, insect, and arachnid. Around the center category, the rest of the tables in the database store the information about morphologies (blue area), manipulations (green area), and experiments (red area). The relations between the tables (arrows) ensure a consistent and optimal organization of the data.

**Figure 5:**
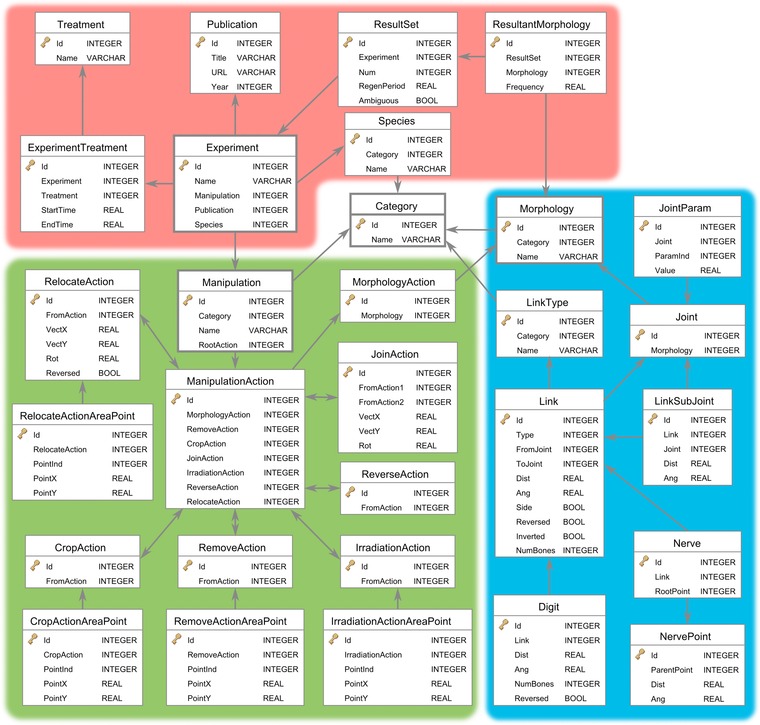
Diagram of the relational database of limb regeneration experiments. The database is organized around organism categories (center) and includes data from experiments (red), morphologies (blue), and manipulations (green).

Following this database schema, we manually curated a centralized database with all the limb regeneration experiments reported in a selection of primary papers from the scientific literature. Table 1 summarizes the publications included in the current version of the database, including their category, species used in the experiments, number of total experiments, average penetrance (the mean number of different phenotypes obtained per experiment and regenerative time) and the type of manipulative actions used (cuts, intralimb and extralimb grafts, pharmaceutical treatments, and irradiations).

**Table 1:** Summary of publications currently included in the database

Reference	Category	Species	#exp.	Average penetrance	Cuts	Intralimb grafts	Extralimb grafts	Pharmac. treat.	Irrad.
Singer and Craven 1948	Salamander	*Notophthulmus viridescens*	13	1.10	X				
Goss 1956a	Salamander	*Triturus viridescens*	2	1.00		X			
Goss 1956b	Salamander	*Triturus viridescens*	10	1.40		X			
Bodemer 1958	Salamander	*Triturus viridescens*	1	2.00	X			X	
Dent 1962	Frog	*Xenopus laevis*	5	3.00	X				
Drachman and Singer 1971	Salamander	*Notophthulmus viridescens*	7	1.43	X			X	
Bryant and Iten 1974	Salamander	*Notophthulmus viridescens*	20	1.35	X				
Stocum 1975	Salamander	*Ambystoma maculatum*	25	2.28	X	X	X		
Iten and Bryant 1975	Salamander	*Notophthulmus viridescens*	32	3.06	X		X		
Smith and Wolpert 1975	Salamander	*Ambystoma mexicanum*	2	1.00	X				
Carlson 1975	Salamander	*Ambystoma mexicanum*	17	1.65	X	X	X	X	
French et al. 1976	Salamander	*Notophthulmus viridescens*	10	1.00	X	X	X		
	Insect	*Blatella germanica*							
Mescher 1976	Salamander	*Notophthulmus viridescens*	2	3.00		X			
Bryant 1976	Salamander	*Notophthulmus viridescens*	6	1.50	X	X	X		
French 1976b	Insect	*Blatella germanica*	5	5.40	X		X	X	
Bohn 1976	Insect	*Blaberus craniifer*	1	3.00	X		X	X	
French 1976a	Insect	*Blatella germanica*	2	5.00	X		X		
Carlson 1977	Salamander	*Notophthulmus viridescens*	23	1.83	X	X	X		X
Bryant and Iten 1977	Salamander	*Notophthulmus viridescens*	15	3.07	X	X	X		
Bryant and Baca 1978	Salamander	*Notophthulmus viridescens*	12	1.92	X	X	X		
Tank 1978	Salamander	*Ambystoma mexicanum*	4	1.75	X	X	X		
Maden and Turner 1978	Salamander	*Ambystoma mexicanum*	4	3.25		X			
Tank and Holder 1978	Salamander	*Ambystoma mexicanum*	12	3.08	X		X		
Maden 1979	Salamander	*Ambystoma mexicanum*	5	2.60	X				X
Stocum 1979	Salamander	*Ambystoma maculatum*	1	1.00	X				
Maden 1980	Salamander	*Ambystoma mexicanum*	5	1.60	X		X		X
Thoms and Fallon 1980	Salamander	*Ambystoma maculatum*	16	3.25	X		X	X	
		*Ambystoma mexicanum*							
		*Notophthulmus viridescens*							
Slack 1980	Salamander	*Ambystoma mexicanum*	46	1.43	X		X	X	
Holder et al. 1980	Salamander	*Ambystoma mexicanum*	8	3.38	X	X	X		
Tank 1981	Salamander	*Ambystoma mexicanum*	3	3.00		X			
Mittenthal 1981	Crustacean	*Procambarus clarkii*	7	2.43	X	X	X		
Randall 1981	Arachnid	*Latrodectus variolus*	12	1.50	X				
Tsonis and Eguchi 1981	Salamander	*Triturus pyrrhogaster*	14	3.14	X			X	
Maden 1982	Salamander	*Ambystoma mexicanum*	17	2.29	X			X	
Maden and Mustafa 1982	Salamander	*Ambystoma mexicanum*	5	3.80	X		X		
Tank 1982	Salamander	*Ambystoma mexicanum*	8	2.75	X		X		
Meinhardt 1983	Salamander	*Notophthulmus viridescens*	4	1.00	X		X		
Lheureux 1983	Salamander	*Pleurodeles waltl*	18	1.06	X		X	X	X
Alberch and Gale 1983	Salamander	*Ambystoma mexicanum*	5	2.20	X			X	
Mittenthal and Trevarrow 1983	Crustacean	*Procambarus clarkii*	5	5.00	X		X		
Mittenthal and Trevarrow 1984	Crustacean	*Procambarus clarkii*	2	5.50	X		X		
Holder and Weekes 1984	Salamander	*Ambystoma mexicanum*	2	3.00	X		X		
Brockes 1987	Salamander	*Notophthulmus viridescens*	2	1.00	X				
Mittenthal 1987	Crustacean	*Procambarus clarkii*	2	3.00	X		X		
Crawford and Stocum 1988b	Salamander	*Ambystoma mexicanum*	8	2.25	X		X	X	
Crawford and Stocum 1988a	Salamander	*Ambystoma mexicanum*	12	2.33	X		X	X	
Egar 1988	Salamander	*Ambystoma mexicanum*	16	1.75	X	X	X	X	
Filoni and Paglialunga 1990	Frog	*Xenopus laevis*	15	1.43	X				
Filoni et al. 1991	Frog	*Xenopus laevis*	7	1.86	X	X	X		
Kao and Chang 1996	Crustacean	*Cancer gracilis*	18	2.72	X	X	X		
		*Cancer productus*							
Jenkins et al. 1996	Salamander	*Notophthulmus viridescens*	2	1.50	X				
Imokawa and Yoshizato 1997	Salamander	*Cynops pyrrhogaster*	3	1.67	X	X	X		
Kao and Chang 1997	Crustacean	*Cancer gracilis*	15	3.00	X	X	X	X	
		*Cancer jordani*							
Christen and Slack 1997	Frog	*Xenopus laevis*	4	1.17	X				
Yokoyama et al. 1998	Frog	*Xenopus laevis*	10	1.00	X		X	X	
Nakatani et al. 1998	Crustacean	*Procambarus clarkii*	3	2.60	X			X	
Kao and Chang 1999	Crustacean	*Cancer gracilis*	10	4.00	X	X	X	X	
		*Cancer productus*							
Nakatani and Kitahara 1999	Crustacean	*Procambarus clarkii*	4	1.50	X			X	
Filoni et al. 1999	Frog	*Xenopus laevis*	7	1.00	X		X	X	
Wang et al. 2000	Salamander	*Notophthulmus viridescens*	9	1.56	X		X	X	
Nakatani 2000	Crustacean	*Procambarus clarkii*	35	1.60	X		X	X	
Roy et al. 2000	Salamander	*Ambystoma mexicanum*	11	1.55	X			X	
Yokoyama et al. 2000	Frog	*Xenopus laevis*	5	4.00	X		X	X	
Yokoyama et al. 2001	Frog	*Xenopus laevis*	6	2.83	X			X	
Roy and Gardiner 2002	Salamander	*Ambystoma mexicanum*	4	1.00	X			X	
Yokoyama et al. 2002	Frog	*Xenopus laevis*	6	1.00	X		X	X	
Endo et al. 2004	Salamander	*Ambystoma mexicanum*	6	1.67	X	X	X		
Echeverri and Tanaka 2005	Salamander	*Ambystoma mexicanum*	2	4.00	X			X	
Kawakami et al. 2006	Salamander	*Ambystoma mexicanum*	1	2.00	X			X	
Hutchison et al. 2007	Salamander	*Ambystoma mexicanum*	2	1.00	X				
Lévesque et al. 2007	Salamander	*Ambystoma mexicanum*	5	1.00	X			X	
Satoh et al. 2007	Salamander	*Ambystoma mexicanum*	1	1.00	X	X	X		
Kumar et al. 2007	Salamander	*Notophthulmus viridescens*	2	1.00	X			X	
Nakamura et al. 2007	Insect	*Gryllus bimaculatus*	8	1.00	X		X	X	
Napiórkowska et al. 2007	Arachnid	*Tegenaria atrica*	3	1.00	X				
Stopper and Wagner 2007	Salamander	*Ambystoma mexicanum*	6	1.67				X	
Yokoyama et al. 2007	Frog	*Xenopus laevis*	6	3.33	X			X	
Stueckle et al. 2008	Crustacean	*Uca pugnax*	14	2.00	X			X	
Nakamura et al. 2008a	Insect	*Gryllus bimaculatus*	9	1.00	X		X	X	
Nakamura et al. 2008b	Insect	*Gryllus bimaculatus*	3	1.00	X			X	
Bando et al. 2009	Insect	*Gryllus bimaculatus*	72	1.14	X		X	X	
Guimond et al. 2010	Salamander	*Ambystoma mexicanum*	14	1.50				X	
Satoh et al. 2010	Salamander	*Ambystoma mexicanum*	7	2.57		X		X	
Yokoyama et al. 2011	Frog	*Xenopus laevis*	6	1.67	X			X	
Zhu et al. 2012	Salamander	*Ambystoma mexicanum*	4	1.00	X			X	
Lee and Gardiner 2012	Salamander	*Ambystoma mexicanum*	4	1.00	X				
Das and Durica 2013	Crustacean	*Uga Pugilator*	16	1.72				X	
Shimokawa et al. 2013	Salamander	*Ambystoma mexicanum*	1	2.00	X			X	
Bando et al. 2013	Insect	*Gryllus bimaculatus*	8	1.00	X			X	
**89 publications**	**5 categories**	**19 species**	**827**	**1.92**	**80**	**24**	**45**	**45**	**4**

We are maintaining the centralized database of limb experiments, which is freely available on the web (http://limbform.daniel‐lobo.com). We are continuously expanding the database with recent and classic papers. In addition, we invite the community to encode their own published experiments and submit them through the same website for inclusion in the centralized database. After some standard checks for consistency and redundancies, the new published experiments will be rapidly included and distributed in an updated version of the centralized database.

### Software tool

We have designed and implemented a stand‐alone software tool to create, read, and analyze databases of limb regeneration experiments. The software tool is called Limbform (Limb Formalization), and it can be used to work with our curated database of limb experiments as well as with anyone's personal database created with the program or following the presented database schema. Limbform presents a user‐friendly graphical interface that allows any scientist to query, input, and easily search the large database of regeneration experiments, manipulations, and morphologies curated in the centralized database.

The software tool Limbform is freely available on the web (http://limbform.daniel‐lobo.com), and it has been specifically designed to interact with the centralized database of experiments. With the software tool and database together, a researcher can easily access and analyze a large selection of regenerative experiments published in the last century. The search functionality included in the tool facilitates the search of specific experiments across all the experiments included in the database. For example, typing a specific drug or gene name into the search module will list all the experiments (including descriptive names, manipulation procedures, and morphological diagrams) related to the drug or gene from any of the publications included in the database. Numerical queries are also possible, such as searching morphologies with a specific number of segments, joints, or digits. Indeed, the combination of the database and software tool represents a complete expert system of limb regeneration experiments. In addition, a user can also use the software tool to expand the database or create new personal databases containing custom experiments.

## Discussion and Conclusion

We propose here the first ontological formalism to mathematically encode limb regenerative experiments. Based on this formalism, we created the first centralized database of limb regeneration experiments curated from the scientific literature of both vertebrate and invertebrate organisms. To facilitate access to the database, we created a software tool for the creation, mining, and analysis of databases of limb regenerative experiments. While other variants may be developed in the future, it is clear that some such scheme is necessary if bioinformatics tools are ever to be developed to leverage the growing mountain of data into constructivist, useful models. Our system is the first proof‐of‐principle effort in this direction, and is modular so that each aspect can be independently altered and improved upon by future users.

The formalism to encode limb phenotypes and their experimental manipulations is based on mathematical graphs. We did not include in the current formalism information regarding the detailed shape of individual limbs. Instead, a series of parameters define the overall shapes of limb segments, ignoring detailed features that greatly differ even among wild‐type limbs. Indeed, the first comprehensive models of limb regeneration constructed based on our formalism and curated data should focus on getting the correct large‐scale bodyplan anatomy and predicting the outcomes of functional perturbation, regardless of fine‐grain detail and individual variability that can add irrelevant noise to the dataset. In contrast to the textual phenotypic representations of classical ontologies, a mathematical graph unambiguously represents a phenotype at different information levels, including logical (merus vs. carpus), topological (foot connected to shank), and geometrical (length of a segment) information. Graph‐based phenotypes can be mathematically analyzed, compared, and mined, not only by scientists but also by automatic algorithms. This aspect takes the current system beyond a standardized repository of knowledge in this field to a first step towards computer‐assisted search for mechanistic models that explain the functional data. The ability of artificial intelligence tools to access and automatically analyze this dataset will pave the way for the application of new bioinformatics tools to mine the experimental knowledge on limb regeneration. Importantly, our formalism allows the seamless inclusion of regenerative data from very different model systems, allowing scientists and data mining algorithms to look for deep (not just species‐specific) patterns in these data.

We implemented a database schema based on the presented formalism and curated the first centralized database of limb regeneration experiments published in the scientific literature. The database unambiguously stores all the relevant details of the experiments, including publication, species, treatments, manipulations, and the reported statistical phenotype results. However, we found important information missing from some published papers, including the number of animals used, penetrance of the treatments, or ambiguous phenotype descriptions using natural language. In these cases of unknown information, we opted to input default values: *N* = 1 for the number of animals used, uniform distribution for penetrance, and a special “other” morphology for unknown or very ambiguously reported phenotypes. The database comprises a large selection of limb‐regenerating organisms, such as salamanders, frogs, insects, crustaceans, and arachnids, and it is freely available online for download. We are continuously expanding the centralized database and distributing new versions online with the most recent published experiments. As the database grows with the addition of further classic papers and new studies, its power to constrain and inform model‐building efforts will likewise increase.

We designed and implemented the software tool Limbform to facilitate the access and use of the centralized limb regeneration database. Anyone can download the freely available software tool and use it for searching specific experiments, extend the database with new experiments, or create new personal limb regeneration databases from scratch. The software presents an intuitive user interface based on graph diagrams and automatically generated cartoon representations of limb morphologies. Importantly, the included search module can be used to find experiments, manipulations, phenotypes, and publications (including online links to the full text) with any desired characteristic from the hundreds of experiments curated in the database. For example, a user can easily list all leg phenotypes (from any species) regenerated after a treatment with retinoic acid.

The current version of the formalism and database will be extended on several fronts. The main limitation of our current implementation is that it is constrained to two dimensions. Although the current formalism supports morphological segments reversed along a third dimension, the implementation of precise rotations will require the full support of three‐dimensional data. The mathematical representation based on graphs can be easily extended to handle three‐dimensional phenotypes, but will require implementation of new intuitive user interfaces for the visualization and manipulation of three‐dimensional morphologies. However, morphological data reported in the literature currently lack three‐dimensional information, due precisely to the lack of adequate tools for the processing and formalization of three‐dimensional phenotypes. Finally, the results stored in the database are currently limited to morphological outcomes; hence, the database does not include information regarding gene expression and protein patterns, genetic sequences, or microscopic tissue details from micrographs that may have been reported along with the experiments. Future versions of the formalism and database can gradually incorporate more and more detailed information and references to existing genetic databases as soon as the data‐driven models become robust enough to deal with fine‐grain morphological details.

Together, the presented formalism, centralized database, and software tool represent a powerful instrument for mining the extensive limb regeneration literature. The ever‐increasing amount of information disseminated throughout the scientific literature is currently encoded in natural language, photographs, and cartoon diagrams of diverse styles, which hinder true insight into mechanistic models of high‐level pattern regulation. Our formalism and database represent the first steps to systematically and unambiguously curate the current knowledge of limb regeneration, facilitating the development of mechanistic models explaining the full extent of the known results and improving the understanding of large‐scale patterning of complex structures. However, this is just the first step in our roadmap for a bioinformatics of shape.

Most importantly, the presented formalism and database is not only an indispensable tool for human scientists but, due to its mathematical nature, can be accessed and interpreted by automated algorithmic methods. Already, the data on limb regeneration are so vast that it is practically impossible to simply think up mechanistic models consistent with all functional data. Assistance from bioinformatics tools will become indispensable in most developmental and regenerative studies. Indeed, regenerative biology will benefit enormously from the application of computational modeling and simulation techniques to find candidate models (Iber and Zeller 2012) using the presented formalism and database of experiments. A complete formalization of experimental knowledge in limb regeneration, our system presented here being a solid foundation, will pave the way to applying algorithms for the automatic search of scientific discoveries (King et al. 2009; Sparkes et al. 2010).

Figure 6 shows a use case diagram of the presented formalization and database, including both human scientists and high‐performance computers. Human scientists can use the database to curate, centralize, and mine the repository of published experiments. In addition, high‐performance computers could simulate mechanistic models of regeneration and test their validity against the experiments stored in the centralized database. We are currently working on artificial intelligence tools that can discover mechanistic models of pattern regeneration, simulate these models in virtual cells, and use the database to determine whether the models correctly predict and explain the body of known data in limb regeneration.

**Figure 6:**
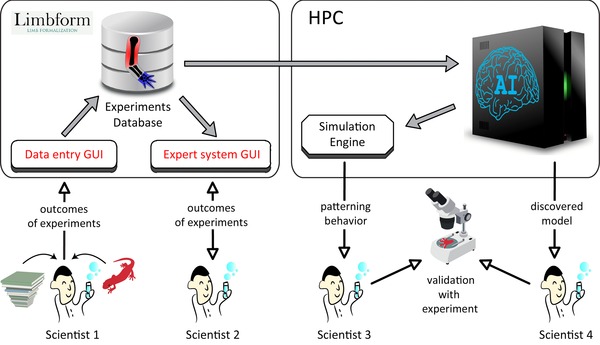
Use case diagram of our envisioned system, with the formal database presented here as the keystone. Researchers can easily and unambiguously curate into the centralized database the outcome of experiments performed in the laboratory and published in the literature (scientist 1). The centralized repository can be queried with an expert system interface to mine the knowledge from hundreds of experiments published in the literature (scientist 2). The database is encoded with a mathematical language, which allows its access by high‐performance (HPC) automated algorithms and simulation engines that can be used to test mechanistic models of patterning and shape formation (scientist 3). Furthermore, artificial intelligence tools can automate the discovery of models that explain the experiments encoded in the database, which can then be validated with further experiments in the laboratory (scientist 4).

In summary, we have presented the beginnings of a roadmap for a new bioinformatics of shape for limb regeneration experiments applicable to many model organisms. Our system is thus a candidate for standardizing the work in this field to facilitate dissemination and comparative analyses of results. Our mathematical approach for encoding and storing experimental, manipulative, and phenotypical information not only will help scientists in the field to access this vast amount of knowledge, but will allow the development of novel bioinformatics tools to help discover testable, mechanistic models from functional perturbation data.

## Materials and Methods

### Database curation

Currently, the centralized database contains the details of 827 different experiments manually curated from 89 publications from the scientific literature and comprising hundreds of manipulations and morphologies (Table 1). The curation process of the centralized database was performed manually with the help of the software tool Limbform. We selected the most important work on limb regeneration from an extensive set of organisms, including salamanders, frogs, crustaceans, insects, and arachnids. Starting with the publications with the highest number of citations, we curated those experiments with altered morphological outcomes. Our selection includes limb regeneration experiments under a variety of manipulative operations such as cuttings, graftings, irradiations, and pharmacological treatments.

### Database implementation

We used the database engine SQLite version 3 (public domain) for implementing the database schema designed for the presented formalism (Fig. 5). SQLite is the most used embedded relational database management system and implements most of the Structured Query Language (SQL), facilitating the interoperability with most of the current database infrastructures. An advantage of SQLite is that the database is contained in a single file, which includes both the schema and the data. This facilitates the administration and sharing of databases, since the database file can be easily copied, downloaded from the web, or sent by e‐mail to a collaborator.

### Software implementation

We implemented the software tool Limbform as a native standalone desktop application for the Microsoft Windows, Mac OS X, and Linux platforms. The tool can create, read, and write databases following the presented scheme for limb regenerative experiments. Limbform stores a database as a single computer file, facilitating the sharing of databases among the research community. The curated centralized database of limb experiments can be downloaded as a single file and be opened for analysis or extension with Limbform.
